# Mechanisms of permselectivity of connexin hemichannels to small molecules

**DOI:** 10.1016/j.jbc.2025.110858

**Published:** 2025-10-24

**Authors:** Alexandra Lovatt, Jack Butler, Nicholas Dale

**Affiliations:** School of Life Sciences, The University of Warwick, Coventry, United Kingdom

**Keywords:** connexins, hemichannels, ATP, glutamate, lactate, N terminus, permeability

## Abstract

Connexins can act either as hemichannels to facilitate ion and small-molecule movement from the cytosol to the extracellular space or as gap junction channels to provide a pathway for solute exchange between cells. Connexins are ubiquitously expressed throughout the body and are implicated in a wide range of processes. The permselectivity of connexin hemichannels for small neurochemicals remains poorly understood. By coexpressing genetically encoded fluorescent sensors for ATP, glutamate, and lactate with a range of connexins, we examined the ability of different hemichannels to permit the release of these compounds under physiological conditions and in response to physiological stimuli (small changes in partial pressure of CO_2_ and transmembrane depolarization). We found that some connexin hemichannels were relatively nonselective (Cx26, Cx32, Cx43, and Cx31.1) allowing passage of ATP, glutamate, and lactate. By contrast, other connexin hemichannels (Cx36, Cx46, and Cx50) were highly selective. Cx36 and Cx46 hemichannels allowed the release of ATP but not glutamate or lactate. The size of the permeating molecule cannot be the sole determinant of permselectivity. By contrast, Cx50 hemichannels permitted the release of lactate and glutamate but not ATP. We also found that the nature of the opening stimulus could alter the permselectivity of the hemichannel—for some of the relatively nonselective connexins, hemichannel opening *via* depolarization was ineffective at allowing the release of lactate. By performing a mutational analysis, informed by the differential selectivity of the closely related Cx46 and Cx50 hemichannels, we found that the charge on the N terminus and N terminus–transmembrane 2 interactions are key contributors to permselectivity for ATP.

There are 21 connexin genes in the human genome (1). Connexins form hexamers that, if unopposed, can act as a plasma membrane hemichannel that opens to the extracellular space. However, hemichannels of closely apposed cells can also dock together to form gap junction channels to provide an aqueous passageway between cells. The structure of the hemichannel is highly conserved in all 21 isoforms; each connexin has an N-terminal helix, four transmembrane (TM) helices, a cytoplasmic loop, two extracellular loops, and a cytoplasmic C terminus (2, 3, 4, 5, 6, 7, 8). Six subunits then coassemble to form a hemichannel with a central pore, spanning ∼1.2 nm that is permeable to small molecules up to a molecular weight of about 1000 Da. Major differences in structure between isoforms lie within the cytoplasmic loop and C terminus, which vary in sequence and length (9). The six N-terminal helices line the hemichannel pore to form the narrowest part of the permeation pathway, suggesting that the N terminus may be important for determining the permeability of the channel (6, 10, 11, 12, 13).

Connexin hemichannels have been documented to release small molecules such as ATP under physiological conditions (14, 15, 16, 17, 18). Yet the mechanisms that control hemichannel permeability to different molecules and whether there is specificity to which molecules may permeate are still unclear. Traditionally, this has been investigated using various fluorescent dyes, such as ethidium bromide, and the size and charge of dyes provided some evidence for selectivity to release of larger molecules (19, 20, 21). Investigation of hemichannel permeability *via* dye fluxes, while valuable, may differ from how physiological metabolites, such as ATP, glutamate, or lactate, permeate these channels. Traditionally, connexin permeability studies have used the removal of extracellular divalent cations to unblock the hemichannels (10, 22). The mechanism was defined in Cx26 and Cx32 and involves a ring of 12 aspartate residues within the extracellular loop that provide a carboxylate cluster able to bind Ca^2+^ ions with millimolar affinity (23, 24, 25). While hemichannels are essentially blocked at Ca^2+^ concentrations over 1 mM, there are very few, if any, physiological conditions in which extracellular Ca^2+^ is lower than 1 mM. Thus, unblocking of hemichannels *via* Ca^2+^ removal may open a permeation pathway that is not representative of physiological gating of connexin hemichannels. Nevertheless, this method has been used to suggest differential permeability of Cx30 and Cx43 hemichannels to a variety of small molecules (10, 22).

Connexin hemichannels can be opened under physiological conditions by other gating stimuli. As the N termini of connexins have charged residues and are within the membrane electric field, almost all connexin hemichannels can be opened by sufficient depolarization, without the need to lower extracellular Ca^2+^ (26). A subset of connexins is directly sensitive to the concentration of gaseous CO_2_ and can be opened by relatively small changes in the partial pressure of CO_2_ (PCO_2_) around the physiological norm. CO_2_-dependent gating was elucidated in Cx26 (27): K125 is carbamylated by CO_2_, which facilitates the formation of a salt bridge with R104 of the adjacent subunit to bias the channel into an open conformation. This mechanism involves movements of the N terminus, along with related movements of the TM helices (particularly TM2) (2, 3). CO_2_ gating has subsequently been discovered in a subset of connexins: Cx30, Cx32 (28), Cx43 (29) and Cx50, since they all have the carbamylation motif that is required for CO_2_-mediated opening. Their CO_2_ sensitivity lies close to the physiological range of PCO_2_; Cx26, Cx43, and Cx50 hemichannels are maximally open at ∼55 mm Hg PCO_2_ and Cx32 at ∼70 mm Hg. All these hemichannels are essentially shut at a PCO_2_ of 20 mm Hg.

To permit the study of the permeation of connexin hemichannels for physiological molecules under their normal electrochemical gradients and with physiological gating stimuli (voltage and PCO_2_), we have developed an assay to allow real-time imaging of analyte release at single-cell resolution. We utilized the genetically encoded sensors GRAB_ATP_ (30), iGluSnFR (31), and eLACCO1.1 (32) to measure the release of ATP, glutamate, and lactate, respectively, *via* coexpressed connexin hemichannels. We find that connexins can be divided essentially into relatively nonselective and highly selective categories. Connexin hemichannels such as those formed by Cx26, Cx32, and Cx43 fall into the relatively nonselective category, whereas hemichannels composed of Cx36, Cx46, and Cx50 are highly selective. By comparing the highly homologous Cx46 and Cx50, we have shown that key residues in the N terminus and in the interacting portion of TM2 determine the permeability profile of the hemichannel.

## Results

**Figure 1:**
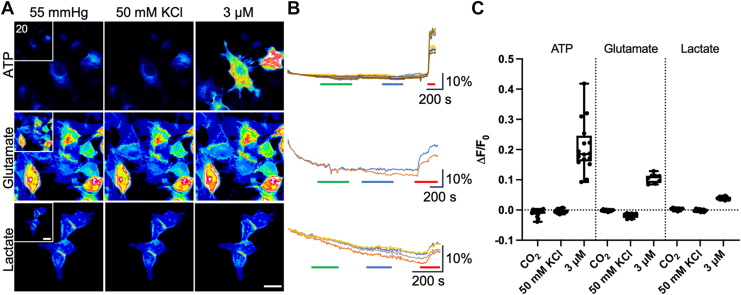
**HeLa cells expressing the genetically encoded fluorescent sensors GRAB_ATP_, iGluSnFr, and eLACCO1.1 alone do not respond to connexin gating stimuli.***A,* representative images of cells at 55 mm Hg PCO_2_ (*inset* is 20 mm Hg baseline control), 50 mM KCl, and after application of 3 μM corresponding analyte. The scale bar represents 20 μm. *B,* representative traces showing sensor responses to 50 mM KCl (*green bar*), 55 mm Hg (*blue bar*), and 3 μM corresponding analyte (*red bar*). Vertical scale bars represent the percent change in ΔF/F_0_. *C,* summary data showing median ΔF/F_0_ for ATP (n = 16 cells), glutamate (n = 8), and lactate (n = 8). *Box and whisker plots* with superimposed data points, showing the median (*line*), interquartile range (*box*), and range (*whiskers*). PCO_2_, partial pressure of CO_2_.

We first transfected HeLa DH cells with the genetically encoded sensors on their own to ensure that the sensors had no intrinsic responses to CO_2_ or high KCl solutions or that parental HeLa cells exhibited ATP, glutamate, or lactate release in the absence of connexin expression. The median change in normalized fluorescence (ΔF/F_0_) for GRAB_ATP_ was −0.007 (95% confidence interval: 0.0043, −0.013) and −0.004 (0.0017, −0.0064) for 55 mm Hg and 50 mM KCl, respectively. GRAB_ATP_ was functional, as it gave a median response of 0.2 (0.25, 0.16) to 3 μM ATP (Fig. 1). The median change in ΔF/F_0_ for iGluSnFR with 55 mm Hg was 0 (0.0008, −0.0026), for 50 mM KCl, it was −0.014 (−0.011, −0.030), and for 3 μM glutamate, it was 0.1 (0.13, 0.05). Finally, the median ΔF/F_0_ for eLACCO1.1 (modified by insertion into iGluSnFR backbone) was 0.005 (0.0073, −0.0046), −0.002 (0.0048, −0.0069), and 0.04 (0.046, 0.032) for 55 mm Hg, 50 mM KCl, and 3 μM lactate, respectively. The negative recorded values are an artifact of photobleaching. As there were no responses from any of the genetically modified sensors to a change in PCO_2_ or membrane depolarization, we conclude that parental HeLa DH cells do not express any channels capable of releasing ATP, glutamate, or lactate to these stimuli.

### Cx26, Cx32, Cx43, and Cx31.3 hemichannels are permeable to ATP, glutamate, and lactate

**Figure 2:**
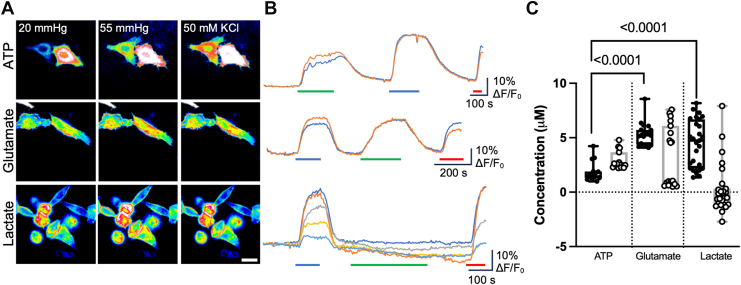
**Cx26 hemichannels mediate release of ATP, glutamate, and lactate.***A,* representative images showing cells from each sensor under each condition. The scale bar represents 20 μm. *B,* representative traces of normalized fluorescence changes in response to 55 mm Hg PCO_2_ (*blue bar*), 50 mM KCl (*green bar*), and a 3 μM calibration of the corresponding analyte (*red bar*). *C,* summary data showing the median release for ATP (n = 15 cells), glutamate (n = 19), and lactate (n = 27) through Cx26 hemichannels stimulated either by 55 mm Hg (*filled circles*) or 50 mM KCl (*open circles*). Kruskal–Wallis ANOVA *p* < 0.0001 for 55 mm Hg CO_2_ and *p* < 0.0001 for 50 mM K^+^. Mann–Whitney *U* tests, *p* < 0.0001 (55 mm Hg ATP *versus* glutamate, 55 mm Hg ATP *versus* lactate). The order of stimulus (CO_2_ or KCl) was regularly reversed between recordings to avoid any potential depletion of ATP release. Data present are from at least three independent transfections. *Box and whisker plots* with superimposed data points, showing the median (*line*), interquartile range (*box*), and range (*whiskers*). PCO_2_, partial pressure of CO_2_.

We next cotransfected HeLa cells with Cx26, Cx32, Cx43, or Cx31.3 and one of the genetically encoded fluorescent sensors. Throughout all the assays, we selected cells that coexpressed the connexin and the sensor for measurement and analysis (Fig. S1). Cx26 hemichannels are highly permeable to ATP, glutamate, and lactate (Fig. 2). The median ATP release to hypercapnic stimuli was 1.5 μM (1.91, 1.32), and with a depolarizing stimulus, it was 2.5 μM (3.67, 2.30). This was significantly less than for glutamate and lactate: the median glutamate release was 5.2 μM (5.71, 4.36) and 1.1 μM (6.10, 0.74) when opened with hypercapnia and a depolarizing stimulus, respectively. Lactate release evoked by hypercapnia was comparable to glutamate: median release of 4.7 μM (6.26, 2.52). However, when stimulated by depolarization, no lactate release was evident: median −0.1 μM (0.23, −1.04). For voltage-gated glutamate release, it appeared that in some cells the depolarizing stimulus was ineffective at evoking release. This suggests that KCl-evoked depolarization (estimated to be about 70 mV from the Nernst equation) is a less reliable gating mechanism than CO_2_ for Cx26.

**Figure 3:**
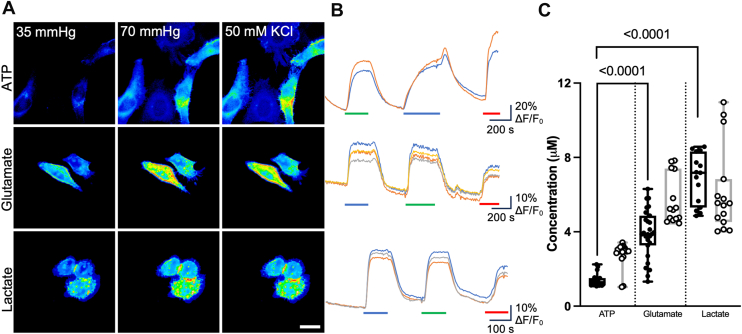
**Cx32 hemichannels mediate release of ATP, glutamate, and lactate.***A,* representative images showing cells from each sensor under each condition. The scale bar represents 20 μm. *B,* representative traces of normalized fluorescence changes in response to 70 mm Hg PCO_2_ (*blue bar*), 50 mM KCl (*green bar*), and a 3 μM calibration of the corresponding analyte (*red bar*). *C*, summary data showing the median release of ATP (n = 18), glutamate (n = 27 for CO_2_, n = 14 for KCl), and lactate (n = 15), through Cx32 hemichannels showing changes in release depending on whether the channel was opened by CO_2_ (*filled circles*) or a depolarizing stimulus (*open circles*). Data presented are from at least three independent transfections. Kruskal–Wallis ANOVA *p* < 0.0001 for 55 mm Hg CO_2_ and *p* < 0.0001 for 50 mM K^+^. Mann–Whitney *U* tests, *p* < 0.0001 (CO_2_ ATP *versus* glutamate), *p* < 0.0001 (CO_2_ ATP *versus* lactate), *p* < 0.0001 (50 mM KCl ATP *versus* glutamate), and *p* < 0.0001 (50 mM KCl ATP *versus* lactate). *Box and whisker plots* with superimposed data points, showing the median (*line*), interquartile range *(box*), and range (*whiskers*). PCO_2_, partial pressure of CO_2_.

We determined that Cx32 hemichannels were also permeable to all three tested analytes. Significantly, more glutamate and lactate were released compared with ATP regardless of the stimulus (Fig. 3). However, the nature of the stimulus did alter the relative amounts of release of the three metabolites. The median release of ATP was 1.3 (1.51, 1.16) and 3.0 (3.12, 2.86) μM when stimulated by hypercapnia and voltage, respectively. Glutamate released *via* Cx32 hemichannels by hypercapnia and depolarization was 3.9 (4.64, 3.41) μM and 5.2 (7.44, 4.62) μM, respectively. Lactate release *via* Cx32 hemichannels by hypercapnia and depolarization was 7.2 (8.32, 5.31) μM and 5.5 (6.84, 4.52) μM, respectively. With hypercapnia, the relative release of both glutamate and lactate compared with ATP was considerably more than might be expected from the calculated electrochemical driving force (Tables 1 and 2). This suggests that Cx32 hemichannels may have enhanced permeability for these molecules when opened by hypercapnia.

**Table 1:** Calculations of the Nernstian equilibrium potential for ATP, glutamate, and lactate in HeLa cells

Analyte	Valence	[Analyte]_I_ (mM)	[Analyte]_o_ (mM)	V_eq_ (mV)	Driving force at rest
ATP	−2	10^a^	10^−4^	145	205
Glutamate	−1	20^b^	10^−4^	307	367
Lactate	−1	1^c^	10^−4^	232	292

We assigned the valence of ATP under the assumption that it is chelated to Mg^2+^. Driving force is calculated assuming a resting potential of −60 mV. The intracellular concentrations were taken from studies that investigated HeLa cells: a, (43); b, (44); and c, (45).

**Table 2:** Relative release of ATP, glutamate, and lactate of connexin hemichannels normalized to amount of ATP release

Connexin	ATP	Glu	Lac
Cx26	1	3.5 (0.44)	3.1 (0)
Cx32	1	3.0 (1.73)	5.5 (1.83)
Cx43	1	2.4 (0.4)	2.0 (0)
Cx31.3	1	0.9	1.1
Cx36	1	0	0
Cx46	1	0	0
Cx50	0	1.5 (0) μM	5.8 (3.4) μM

The numbers given are for the hypercapnic stimulus, except for Cx31.3, Cx36, and Cx46, which are insensitive to CO_2_. For those channels sensitive to CO_2_, the numbers in brackets are normalized values for the depolarizing stimulus. For Cx50, which is not permeable to ATP, the numbers are absolute concentration in micromolar. The ratio of the Nernstian driving force for each analyte (normalized to that of ATP) during the hypercapnic stimulus is 1:1.8:1.4, during the depolarizing stimulus it is (1:2.1:1.6) assuming that the membrane depolarizes to 0 mV.

**Figure 4:**
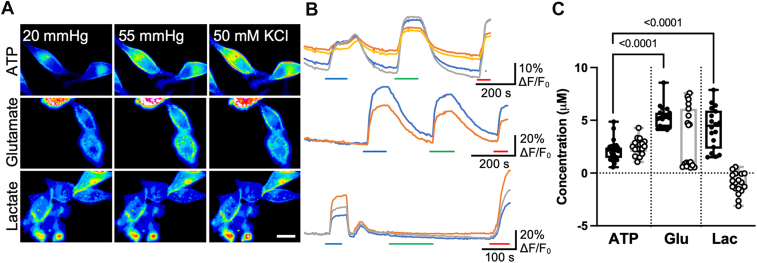
**Cx43 hemichannels mediate the release of ATP, glutamate, and lactate.***A,* representative images showing cells from each sensor under each condition. The scale bar represents 20 μm. *B,* representative traces of normalized fluorescence changes in response to 55 mm Hg PCO_2_ (*blue bar*), 50 mM KCl (*green bar*), and a 3 μM calibration of the corresponding analyte (*red bar*). *C,* summary data showing the median release of analytes through Cx43 hemichannels: ATP (n = 33 (CO_2_), n = 17 (high KCl), glutamate (n = 19), and lactate (n = 18). Data show CO_2_-dependent release (*filled circles*) and release to a depolarizing stimulus (*open circles*). Data presented are from at least three independent transfections. Kruskal–Wallis ANOVA *p* < 0.0001 for 55 mm Hg CO_2_ and *p* < 0.0001 for 50 mM K^+^. Mann–Whitney *U* tests, *p* < 0.0001 (CO_2_ ATP *versus* glutamate), *p* < 0.0001 (CO_2_ ATP *versus* lactate). *Box and whisker plots* with superimposed data points, showing the median (*line*), interquartile range (*box*), and range (*whiskers*). PCO_2_, partial pressure of CO_2_.

With a hypercapnic stimulus, a hemichannel composed of Cx43 was permeable to ATP, glutamate, and lactate (Fig. 4). The median ATP, glutamate, and lactate release were, respectively, 2.2 (2.34, 1.49) μM, 5.3 (5.71, 4.36) μM, and 4.5 (5.84, 2.46) μM (Fig. 4). This amount of release is roughly proportional to the electrochemical driving force on these molecules (Tables 1 and 2), suggesting no selectivity. However, during membrane depolarization evoked by 50 mM KCl, the permeation profile was different. Whereas median ATP release was 2.5 (2.88, 2.22) μM (similar to that evoked by CO_2_), the release of glutamate was reduced, median 1.0 (6.10, 0.74) μM, and lactate was not released at all during this stimulus, median −1.0 (−0.069, −1.49) μM. The gating mechanism of Cx43 thus alters the permeability profile of the hemichannel to small molecules.

While permeability of Cx43 hemichannels to ATP has been elegantly demonstrated (33), previous reports suggest that Cx43 hemichannels are apparently not permeable to glutamate or lactate (10, 22). However, these previous studies used removal of extracellular Ca^2+^ to unblock the channel, and the channel might thus have a different permeability profile. Whereas zero [Ca^2+^]_ext_ is often achieved by the use of chelators, such as EGTA, the genetically encoded fluorescent sensors have some degree of Ca^2+^ dependency. To ensure compatibility with the sensors, we omitted EGTA and Ca^2+^ to lower but not eliminate extracellular Ca^2+^. To ensure this was still sufficient to open the hemichannels, we employed a dye loading assay using the hemichannel-permeable dye, FITC. Under control conditions (PCO_2_ 20 mm Hg, 2 mM [Ca^2+^]_ext_), the median pixel intensity was 15.7 (24.72, 14.04). In low [Ca^2+^]_ext_ (PCO_2_ 20 mm Hg), the median pixel intensity was 54.3 (86,74, 51.82) (Fig. S2). For a comparison to a reliable opening stimulus, we used a PCO_2_ of 55 mm Hg to open the hemichannels and permit loading with FITC. This yielded a median pixel intensity of 48.0 (54.83, 40.66) and demonstrates that the low [Ca^2+^]_ext_ solution was an effective stimulus to open Cx43 hemichannels.

Having established the efficacy of low [Ca^2+^]_ext_ at opening Cx43 hemichannels, we assessed the permeation of ATP, glutamate, and lactate during this stimulus and compared it with permeation in response to hypercapnia in the same cells. Consistent with our previous findings, hypercapnia evoked the release of ATP, glutamate, and lactate. However, low [Ca^2+^]_ext_ significantly reduced the analyte release through Cx43 hemichannels, with a median release of 0.1 (0.86, 0.08) μM ATP, 0.2 (0.76, 0.004) μM glutamate, and 0.5 (2.11, −0.25) μM lactate (Fig. S3). The nature of the gating mechanism appears to change channel permeability and may account for why our results differ from previous release studies.

**Figure 5:**
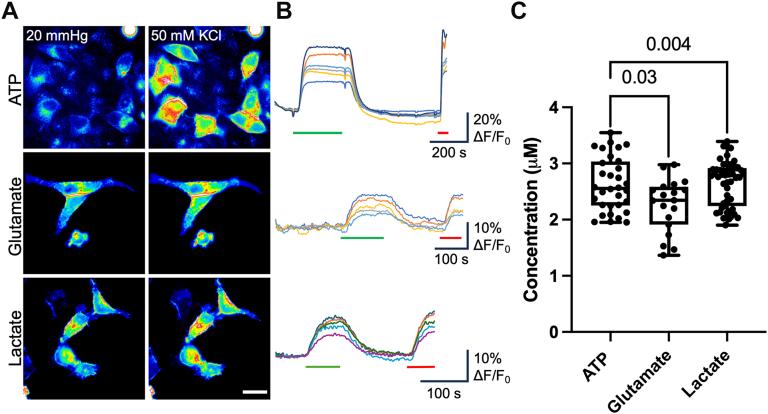
**Cx31.3 hemichannels mediate release of ATP, glutamate, and lactate.***A,* representative images showing cells from each sensor under each condition. The scale bar represents 20 μm. *B,* representative traces of normalized fluorescence changes in response to 50 mM KCl (*green bar*) and 3 μM analyte (*red bar*). *C,* summary data showing the median ATP (n = 32), glutamate (n = 19), and lactate (n = 50) release from Cx31.3 hemichannels in response to a depolarizing stimulus. Data presented are from at least three independent transfections. Kruskal–Wallis ANOVA *p* = 0.0174. Mann–Whitney *U* tests, *p* = 0.004 (ATP *versus* glutamate) and *p* = 0.03 (lactate *versus* glutamate). *Box and whisker plots* with superimposed data points, showing the median (*line*), interquartile range (*box*), and range (*whiskers*).

Cx31.3 (also called Cx29) is mainly expressed in myelinating cells (34). There has been some suggestion that Cx31.3 predominantly forms hemichannels and mediates ATP efflux from cells that express this isoform (35, 36). As Cx31.3 is not CO_2_ sensitive (37), we used a depolarizing stimulus to open this hemichannel. We were able to demonstrate a median release of 2.6 (2.91, 2.26) μM ATP, 2.3 (2.58, 1.91) μM glutamate, and 2.8 (2.92, 2.24) μM lactate (Fig. 5) *via* Cx31.3 hemichannels. While relatively nonselective, Cx31.3 hemichannels nevertheless show some preference for ATP, as the relative permeation of glutamate and lactate is less than that predicted by the electrochemical driving force on these molecules (Tables 1 and 2).

### Cx36, Cx46, and Cx50 hemichannels have highly specific permeability profiles

**Figure 6:**
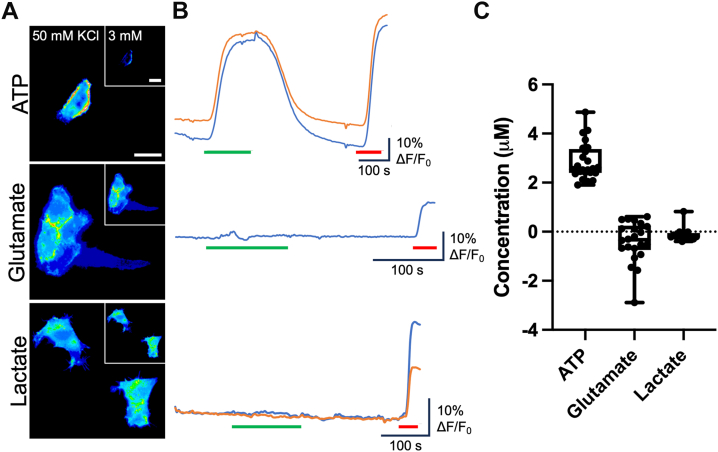
**Cx36 hemichannels mediate release of ATP to depolarization but are impermeable to glutamate and lactate.***A,* representative images showing cells from each sensor under each condition: ATP (n = 24), glutamate (n = 22), and lactate (n = 15). *Inset* is 3 mM KCl baseline control. The scale bar represents 20 μm. *B,* representative traces of normalized fluorescence changes in response to 50 mM KCl (*green bar*) and 3 μM analyte (*red bar*). *C,* summary data depicting ATP release *via* Cx36 hemichannels and no permeability to glutamate or lactate. Kruskal–Wallis ANOVA *p* < 0.0001. Mann–Whitney *U* tests, *p* < 0.0001 (ATP *versus* glutamate, ATP *versus* lactate). *Box and whisker plots* with superimposed data points, showing the median (*line*), interquartile range (*box*), and range (*whiskers*).

Cx36 acts predominantly as a major neuronal connexin (38) and forms the gap junctions or electrical synapses that facilitate fast synaptic transmission and synchronous neuronal firing (39, 40, 41). We assayed the release of ATP, glutamate, and lactate from HeLa cells transfected with Cx36. Because Cx36 is insensitive to CO_2_ (28), we used the depolarizing stimulus to gate the channel. Surprisingly, we found that of the three analytes, Cx36 was only permeable to ATP (Fig. 6). The median release of ATP evoked by 50 mM KCl was 2.6 (3.27, 2.41) μM, compared with −0.3 (0.22, −0.68) and −0.2 (−0.05, −0.21) μM glutamate and lactate, respectively. As the electrochemical driving force for release of glutamate and lactate is about double that of ATP (Tables 1 and 2), and these molecules are smaller than ATP, the differential permeability of Cx36 suggests the existence of a selectivity filter within the pore.

**Figure 7:**
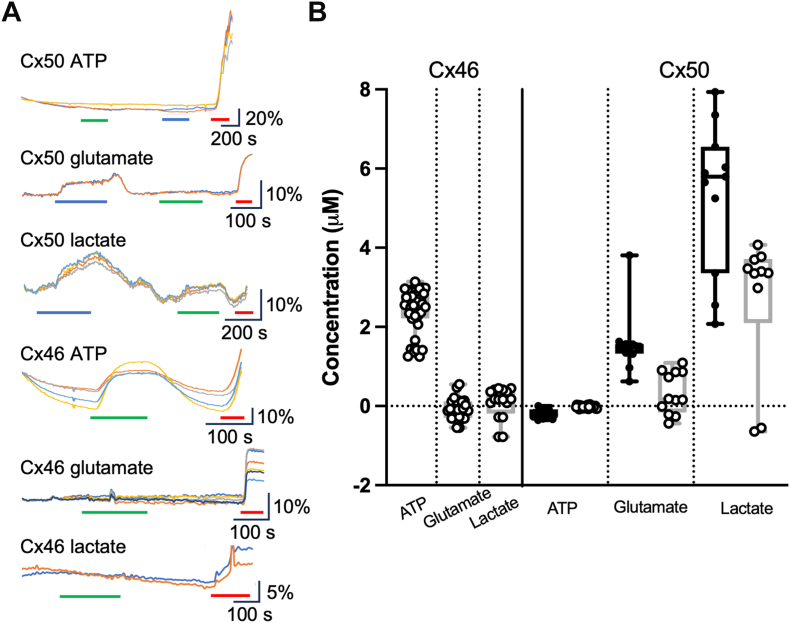
**Cx46 and Cx50 hemichannels have opposing permeability profiles.***A,* representative traces showing normalized fluorescence changes in response to 55 mm Hg (*blue bar*), 50 mM KCl (*green bar*), and 3 μM analyte (*red bar*). *B,* summary data depicting analyte release under each stimulus: Cx46 ATP (n = 40), Cx46 glutamate (n = 25), Cx46 lactate (n = 16), Cx50 ATP (n = 28), Cx50 glutamate (n = 12), and Cx50 lactate (n = 11). Cx50 is CO_2_ sensitive, and thus, release is stimulated by 55 mm Hg PCO_2_ (*filled circles*) and 50 mM KCl (*open circles*). Data presented are from three independent transfections. *Box and whisker plots* with superimposed data points, showing the median (*line*), interquartile range (*box*), and range (*whiskers*). PCO_2_, partial pressure of CO_2_.

Human Cx46 also lacks the carbamylation motif and is not sensitive to CO_2_. We therefore used the high K^+^ stimulus to open Cx46 hemichannels. Like Cx36, Cx46 hemichannels were only permeable to ATP, giving a median release of 2.6 μM (2.74, 2.34) (Fig. 7). No glutamate or lactate was released *via* Cx46 hemichannels (median release −0.07 μM [0.11, −0.28] and 0.2 μM [0.41, −0.28] for glutamate and lactate, respectively, Fig. 7). By contrast, Cx50 hemichannels were readily permeable to glutamate (median release, 1.5 μM [1.57, 1.32]) when stimulated by hypercapnia and lactate when stimulated by either hypercapnia or depolarization (median release, 5.8 μM [7.36, 2.55] and 3.4 μM [3.78, −0.55], respectively, Fig. 7). However, no ATP could permeate Cx50 hemichannels (Fig. 7).

### Mutational analysis of the differential permeability of Cx46 and Cx50 hemichannels

**Figure 8:**
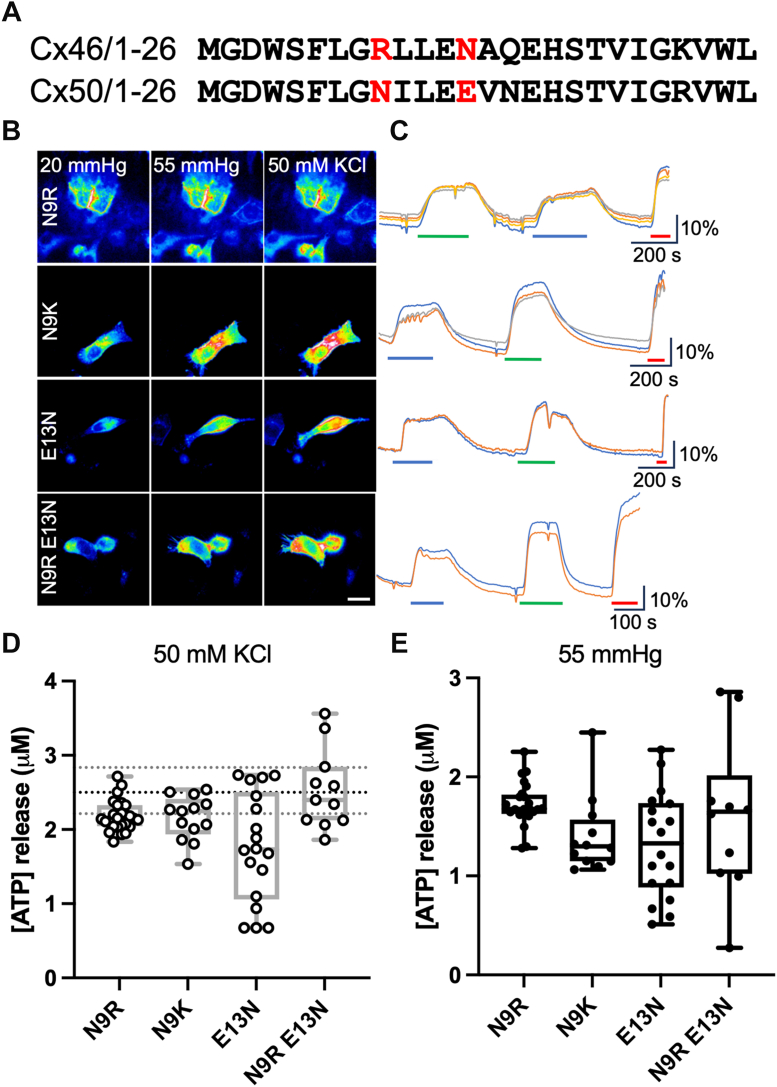
**N-terminal mutations make Cx50 hemichannels permeable to ATP.***A,* sequence alignments for residues 1 to 26 of Cx46 and Cx50 to show the residues in Cx50 that have been mutated to those in Cx46 (*red letters*). *B,* representative images showing cells from each sensor under each condition. The scale bar represents 20 μm. *C,* representative traces of normalized fluorescence changes in response to 55 mm Hg (*blue bar*), 50 mM KCl (*green bar*), and 3 μM ATP (*red bar*). *D,* summary data showing ATP release in response to depolarization from Cx50 mutant hemichannels: N9R (n = 26), N9K (n = 13), E13N (n = 18), and N9R E13N (n = 11). Data show ATP release to a depolarizing stimulus. The *dotted lines* show the median and 95% confidence limits for ATP release from Cx46 in response to depolarization. Kruskal–Wallis ANOVA (all four mutants plus Cx46^WT^) *p* = 0.0009. Mann–Whitney *U* tests, *p* = 0.0018, *p* = 0.0155, and *p* = 0.0004 (N9R, N9K, and E13N, respectively, where release evoked by 50 mM KCl is compared with Cx46^WT^) and *p* = 0.96 (Cx46^WT^*versus* Cx50^N9R E13N^). *Box and whisker plots* with superimposed data points, showing the median (*line*), interquartile range (*box*), and range (*whiskers*). *E,* summary data demonstrating CO_2_-evoked ATP release form the Cx50 mutant hemichannels N9R (n = 26), N9K (n = 13), E13N (n = 18), and N9R E13N (n = 11). *Box and whisker plots* with superimposed data points, showing the median (*line*), interquartile range (*box*), and range (*whiskers*).

Cx46 and Cx50 are structurally quite similar (4, 7, 13), yet their hemichannels have markedly different permeability profiles. These two connexins therefore offer an opportunity to explore the mechanistic basis of differential hemichannel permeability. The N termini fold into the gap junction pore to form the narrowest point, with hydrophobic residues anchoring the helix to TM regions 1/2 to stabilize the open state (7). Aligning the N-terminal sequences of Cx46 and Cx50 shows that they have a difference in the overall net charge (0 and -2 for Cx46 and Cx50, respectively; Fig. 8). The first divergence is at position 9, where Cx46 has a positively charged arginine and Cx50 has an asparagine. At position 13, Cx50 has a negatively charged glutamate residue, where Cx46 has a neutrally charged asparagine.

To explore the possible roles of the difference in net charge, we introduced the single mutations N9R, E13N, and N9K into Cx50 to make it more like Cx46. These mutations gave a gain of ATP permeability to Cx50 hemichannels, though this was still significantly less than that of Cx46^WT^ hemichannels (Fig. 8). The double mutation, Cx50^N9R E13N^, gave an increase in ATP release that matched that of Cx46^WT^ hemichannel release (for a depolarizing stimulus), with a median release of 2.4 (3.37, 2.07) μM from the mutant connexin compared with 2.5 (2.74, 2.34) for Cx46^WT^ hemichannels. We have therefore shown that the net charge of the N terminus and the specific residues N9 and E13 act to regulate the ATP permeability of Cx50 hemichannels.

**Figure 9:**
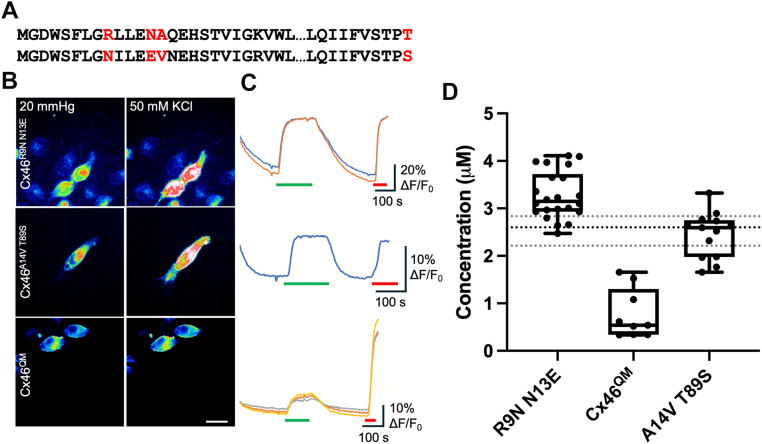
**The permeability of Cx46 hemichannels to ATP appears to be reliant on N-terminal charge and interactions between the N terminus and TM2.***A,* sequence alignments from 1 to 26 and 80 to 89 for Cx46 (*top*) and Cx50 *(bottom*) to show the residues in Cx46, which have been mutated to correspond to those in Cx50 (*red*). *B,* representative images showing cells expressing Cx46^R9N N13E^, Cx46^A14V T89S^, and Cx46^QM^ under each condition. The scale bar represents 20 μm. *C,* representative traces showing normalized change in fluorescence to 50 mM KCl (*green bar*) and 3 μM ATP (*red bar*). *D,* summary data depicting the median ATP release from hemichannels of Cx46^WT^ (the *dotted lines* show the median and 95% confidence interval for Cx46^WT^), Cx46^R9N N13E^ (n = 22), Cx46^QM^ (n = 9), and Cx46^A14V T89S^ (n = 14). Mann–Whitney *U* test, *p* < 0.0001 (Cx46^WT^*versus* Cx46^QM^). *Box and whisker plots* with superimposed data points, showing the median (*line*), nterquartile range (*box*), and range (*whiskers*). TM2, transmembrane 2.

We next examined whether, if we made the N terminus of Cx46 more like that of Cx50, we could change the permeability profile of Cx46 hemichannels to match those of Cx50. We therefore made the double mutation R9N,N13E in Cx46. However, this did not diminish ATP permeation *via* Cx46 hemichannels (Fig. 9). The median release from Cx46^R9N,N13E^ hemichannels was 2.8 (3.10,2.47), compared with 2.5 (2.74, 2.34) μM for Cx46^WT^ hemichannels. This indicates that the regulation of permeability of Cx46 hemichannels to ATP is more complex than just the net charge of the N terminus.

We considered the possibility that interactions between the N terminus and TM2 may differ between Cx46 and Cx50, and this could permit ATP permeation even if the net charge of the N termini was negative. Both Cx46 and Cx50 have hydrophobic residues at position 14: Cx50 has a valine, but Cx46 has an alanine. We therefore used experimentally determined structures of these connexins (42) to identify possible interacting residues in TM2. This highlighted residue 89 as potentially important: in Cx50, this is serine, but in Cx46, it is threonine. We sought to make these interactions in Cx46 more similar to those occurring in Cx50 by introducing the double mutation A14V, T89S. We note that simple introduction of the Cx50 N-terminal helix into Cx46 has been reported as resulting in nonfunctional gap junction channels because of a steric clash between V14 and T89 in the chimeric channel (13). By making two mutations in Cx46, A14V, and T89S, we have avoided this clash. The double mutation A14V, T89S did not by itself alter ATP permeation (Fig. 9), indicating that the Cx46^A14V,T89S^ hemichannel gated normally to voltage. However, when A14V and T89S were then combined with R9N and N13E, the quadruply mutated Cx46^R9N,N13E,A14V,T89S^ hemichannels (Cx46^QM^) exhibited significantly reduced ATP permeation compared with the wildtype hemichannels, with a median release of 0.54 (1.53, 0.34) μM (Fig. 9).

**Figure 10:**
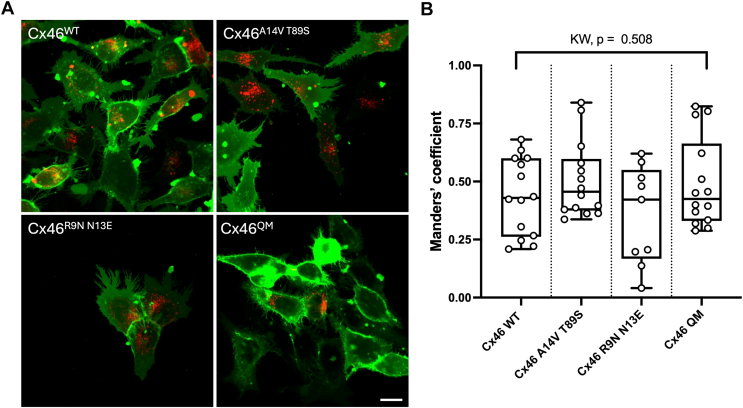
**The plasma membrane distribution of Cx46 is unaltered by mutations that alter its permeability to ATP.***A,* confocal images of DiO staining (*green*) and the mCherry-tagged Cx46 expression (single optical plane). The scale bar represents 15 μm. *B,* Manders' analysis of colocalization shows that the proportion of mCherry colocalized with DiO is the same for Cx46^WT^ and the three mutated variants. Data are from three independent transfections. Kruskal–Wallis ANOVA, *p* = 0.508. DiO, 3,3′-dioctadecyloxacarbocyanine perchlorate.

To check that the mutations of Cx46 and Cx50 did not alter the trafficking and membrane localization of the resulting hemichannels, we performed confocal analysis to quantify the colocalization of the mCherry tag with a plasma membrane stain 3,3′-dioctadecyloxacarbocyanine perchlorate (DiO). Using Manders' analysis, we found that the proportion of mCherry colocalized with DiO was the same in all mutations and their respective wildtype connexins (Figs. 10 and S4).

**Figure 11:**
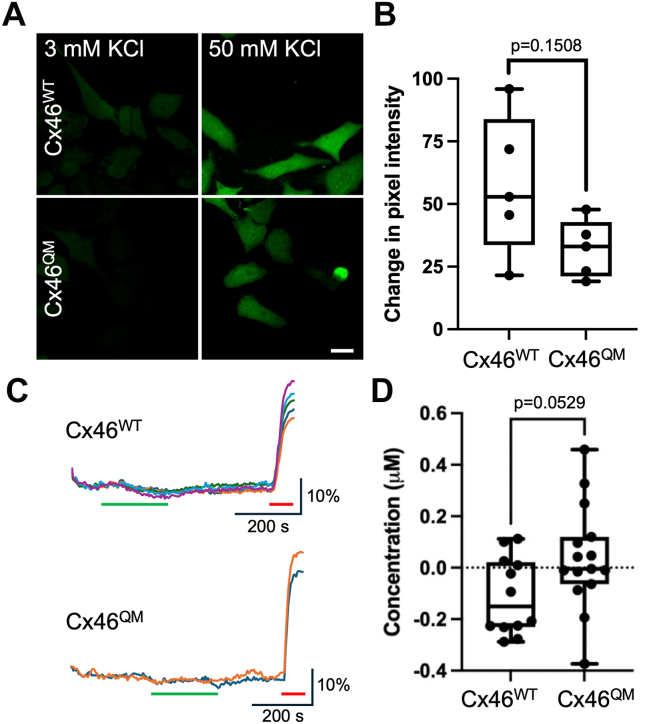
**Cx46^QM^ hemichannels remain permeable to FITC and do not gain permeability to glutamate.***A,* representative images showing cells expressing Cx46^WT^ or Cx46^QM^ loaded with FITC under each condition. The scale bar represents 15 μm. *B,* summary data depicting the median change in fluorescence pixel intensity between 3 mM KCl and 50 mM KCl for cells expressing Cx46^WT^ or Cx46^QM^ from five independent transfections. There is no significant difference in FITC loading into the Cx46^QM^ compared with Cx46^WT^ (Mann–Whitney *U* test). For Cx46^WT^, 43 cells in 20 mm Hg and 35 cells in 50 mM KCl were analyzed. For Cx46^QM^, 79 cells in 20 mm Hg and 79 cells for Cx46^QM^ in 50 mM KCl were analyzed. *Box and whisker plots* with superimposed data points, showing the median (*line*), interquartile range (*box*), and range (*whiskers*). *C,* iGluSnFR traces for Cx46^WT^ and Cx46^QM^ showing that 50 mM KCl (*green bar*) does not evoke glutamate release. *Red bar* 3 μM Glu. *D,* summary data showing median glutamate release from Cx46^WT^ (n = 12) and Cx46^QM^ (n = 15) (three independent transfections, Mann–Whitney *U* test). *Box and whisker plots* with superimposed data points, showing the median (*line*), interquartile range (*box*), and range (*whiskers*).

One possible interpretation of the reduced permeability to ATP of Cx46^QM^ is that the quadruple mutation simply reduces overall hemichannel permeability rather than having a specific effect on that of ATP. To evaluate this, we examined depolarization-dependent loading of FITC into HeLa cells expressing either Cx46^WT^ or Cx46^QM^ (Fig. 11, *A* and *B*). We found that FITC still permeated into cells expressing Cx46^QM^ and was not significantly different from the permeation observed in those expressing Cx46^WT^. This suggests that the quadruple mutant has a somewhat selective effect on ATP permeation. We also checked whether Cx46^QM^ might display enhanced glutamate permeability (as per Cx50 hemichannels). However, this was not the case (Fig. 11, *C* and *D*), suggesting that the determinants of permeation of these two analytes in Cx46 are somewhat independent.

## Discussion

This study explored whether there is selectivity to the release of small molecules from connexin hemichannels expressed in HeLa cells. To assess their relative permeability *via* the different connexins, we need to understand the electrochemical driving force on the three metabolites. Whereas this driving force on each metabolite is likely to be cell type specific, the permeability properties of a connexin hemichannel pore are encoded by the protein and are likely to remain invariant between different cell types. As ATP, glutamate, and lactate are charged, we can use the Nernst equation, along with typical concentrations of these molecules in HeLa cells (43, 44, 45), to calculate the equilibrium potential (Table 1). If we assume that the concentrations of these metabolites are scattered around a mean value that is consistent across all HeLa cells, sufficient recordings of release should statistically reflect these TM concentrations. Based on this analysis, if release through connexin hemichannels were to be nonselective, the relative proportions of release should correspond to the relative electrochemical driving forces. Thus, for a completely nonselective connexin hemichannel, we would expect to see ATP, glutamate, and lactate released approximately in the proportion 1:1.8:1.4 (assuming a resting potential of −60 mV) to hypercapnia and in the proportion 1:2.1:1.6 for the depolarizing stimulus. Any notable deviation from these proportions would indicate that there is some selectivity to the release of small molecules through connexin hemichannels (Table 2).

Our study is the first to produce a comprehensive permeability profile of a wide range of connexin isoforms for release of physiological metabolites with physiological opening stimuli. Connexin hemichannels fall into two broad categories: relatively nonselective (Cx26, Cx32, Cx43, and Cx31.3) and highly selective (Cx36, Cx46, and Cx50). For Cx26 and Cx32 hemichannels, the release of glutamate and lactate relative to ATP is more than predicted by the electrochemical driving force, suggesting that the smaller molecules may permeate more readily than ATP (Table 2). Cx32 hemichannels in particular show enhanced permeability to lactate (nearly four times that predicted by driving force). Interestingly, when Cx32 hemichannels were opened by depolarization, the relative release of analytes followed that predicted by the electrochemical driving force more closely. For Cx43 hemichannels, when opened by hypercapnia, the relative release of ATP, glutamate, and lactate follows very closely the pattern predicted by the electrochemical driving force (Table 2). Cx31.3 hemichannels show some preference for ATP over glutamate and lactate but are nevertheless permeable to these smaller molecules (Table 2).

Rather surprisingly, Cx50 hemichannels are impermeable to ATP. To our knowledge, this is the only connexin hemichannel that ATP cannot permeate. Traditionally, connexin hemichannel permeability studies have studied selectivity by increasing the size of fluorescent dyes (46, 47), with the idea being that any molecule that was below the limiting pore diameter (around 12 Å) should permeate. This would suggest minimal selectivity between permeants, leaving the major driving force as intracellular concentration. Our results modify this idea. Although the smaller molecules glutamate and lactate do permeate the relatively nonselective channels more easily, lactate, being the smallest molecule, should permeate all connexin hemichannels: but it cannot permeate Cx36 or Cx46 hemichannels, whereas ATP, a much larger molecule, can.

We also find that the permeability profile of the hemichannel can alter with the nature of the gating stimulus. For those connexins that are directly CO_2_ sensitive, opening the hemichannel by hypercapnia seems to give greater release than depolarization (summarized in Table 2). One possibility is that 50 mM KCl, while predicted to depolarize the cell by about 70 mV, may not sufficiently depolarize the membrane to obtain full channel opening. We also find that lowered [Ca^2+^]_ext_ seems to be the least effective stimulus for release. While this manipulation may unblock the channel (by removing the ring of bound Ca^2+^ ions), the N termini may still partially block the channel and alter the permeation pathway in a way that may not happen with a more physiological stimulus.

### The selective permeability of connexins may match their physiological roles

That there are 21 connexin genes in the human genome indicates distinct fundamental roles in physiological processes. The need for so many isoforms suggests functional specialization—combinations of properties that match connexin to function. Sensitivity to gating stimuli and permeability to small molecules are two important properties that may determine which functional roles connexins are suited to.

The relatively nonselective connexins, Cx26, Cx32, and Cx43, have different CO_2_ sensitivity profiles. Cx26 is suited to the detection of systemic PCO_2_ and has a role in the control of breathing (15, 17, 48). Cx32 requires much higher levels of PCO_2_ to open and may be more suited to detecting local CO_2_ production (28, 49, 50). It is interesting that Cx32 hemichannels are highly permeable to lactate when opened by hypercapnia. This lends support to an attractive hypothesis that Cx32 hemichannels may detect hotspots of metabolic activity and, by opening and permitting release of lactate, could provide metabolic support for highly active cells (51). Cx43 with its extensive C terminus interacts with many other proteins (52, 53), yet is also CO_2_ sensitive (54) and its hemichannels are partially open under physiological conditions (14, 55).

We also discovered a group of connexins with highly selective hemichannel permeability profiles: Cx36, Cx46, and Cx50. Cx36 is expressed predominantly in neurons, and the hemichannel is impermeable to glutamate and lactate. The lack of glutamate permeability may be functionally significant, as most excitatory neurons use this as their main neurotransmitter. This lack of permeability may ensure that glutamate release is tightly regulated under physiological conditions and occurs mainly *via* vesicular exocytosis at synaptic sites. As lactate is an effective metabolite for oxidative phosphorylation in neurons, the lack of permeability of Cx36 hemichannels to lactate would prevent unregulated efflux of lactate from neurons.

Cx46 and Cx50 are expressed almost exclusively in the lens of the eye (56, 57). Cx46 and Cx50 form gap junctions between the lens epithelial and fiber cells and between lens fiber cells. Cx50 is also present as hemichannels in lens fiber cells. As lens fiber cells mature, they lose their intracellular organelles, including mitochondria (58), and thus the ability to make ATP *via* oxidative phosphorylation. One can speculate that having ATP-permeable Cx46 gap junction channels may be valuable in allowing diffusion of this key metabolite from the metabolically active cells in the lens into the relatively inactive lens fiber cells. Equally, the lack of ATP permeability of Cx50 hemichannels may permit preservation of this scarce resource in the lens fiber cells.

### The N terminus is a fundamental determinant of selectivity

Considerable evidence suggest that the N terminus is part of the gating mechanism of connexins. Depending on the isoform, the N terminus projects into the pore to form an occluding plug or forms a cap at the cytoplasmic vestibule, also to close it. In the open state, the N termini line the pore and thus experience the membrane electric field. This may explain why increasing the charge of the Cx50 N terminus appears to enhance the voltage gating of the mutants (Figs. 7 and 8) (59). As the N terminus lines the entrance to the pore (2, 3, 4, 6, 7, 13, 42), it may regulate selectivity to small molecules. Our evidence suggests that this is the case: by manipulating the charge on the N terminus of Cx50 to make it more like Cx46, we were able to create a mutant hemichannel that gained ATP sensitivity over the wildtype Cx50 hemichannel. We note that the mutation Cx50^N9R^ increased the permeability to ATP through the hemichannel, despite this mutation being reported to decrease the conductance of the Cx50 gap junction channel (13). However, the reverse set of mutations—changing charge on the N terminus of Cx46 to make it more Cx50 like—did not diminish ATP permeability of the mutant Cx46 hemichannel. This shows that other mechanisms can be important.

Our data suggest that residues that alter the interaction between the N terminus and the lining of the pore—notably TM2—play a role in hemichannel selectivity (2, 3). We identified Ala14 and Thr89 of Cx46 as residues that might be important in this interaction and mutated those to their equivalent in Cx50 (A14V, T89S). Thr89 in Cx46 is equivalent to Ala88 of Cx26, which interacts with Val13 on the N terminus (2). Substitution of larger residues (A88V) changes Cx26 channel function and is pathological (60, 61). However, the double mutation in which a smaller residue is substituted (T89S) avoids the steric clash between V14 and T89 previously reported in Cx46-50 chimeric channels, which were nonfunctional (13). The double mutations A14V and T89S or R9N and N13E by themselves did not alter ATP permeability. However, when all four mutations were combined to create Cx46^QM^, there was a considerable reduction of ATP release compared with Cx46^WT^. The effect of the quadruple mutation FITC permeation was not statistically significant. This suggests that rather than simply resulting in a poorly permeable channel, the quadruple mutation has a somewhat selective effect on the ATP permeation pathway. Our study highlights not only the importance of the N terminus in determining selectivity of connexin hemichannels to small molecules but also that even in two very closely related connexins, there are other structural elements that control permselectivity.

## Experimental procedures

### Connexin mutagenesis

Complementary DNAs for the Cx26 and Cx32 genes were synthesized by GenScript and for the Cx46, Cx50, Cx36, Cx43, and Cx31.3 genes by IDT. These were subsequently subcloned into pCAG-GS-mCherry vector prior to transfection. Point mutations were introduced using Gibson assembly. Overlapping fragments both containing the desired mutation were PCR amplified with primers (IDT). Successful mutagenesis was confirmed using Sanger sequencing (GATC Biotech). Double mutants (Cx50^N9R E13N^, Cx46^R9N N13E^) were cloned using successive Gibson assemblies. All Cx constructs were inserted upstream of an mCherry tag, linked *via* a 12 AA linker (GVPRARDPPVAT).

### Cell culture

We used HeLa DH cells as an expression system, as they exhibit very low expression of endogenous connexins and have been used for this purpose by numerous authors over a long period (62, 63). Parental HeLa DH cells (ECACC 96112022; Research Resource Identifier [RRID]: CVCL_2483) were cultured with low-glucose Dulbecco's modified Eagle's medium (Merck Life Sciences UK Ltd; catalog no.: D6046) supplemented with 10% fetal bovine serum (Labtech.com; catalog no.: FCS-SA) and 5% penicillin–streptomycin. The HeLa cells were free from mycoplasma. PCR of cell culture supernatant was regularly performed to check for mycoplasma contamination using the EZ-PCR mycoplasma detection kit (SARTORIUS; catalog no.: 20-700-20). Cells were seeded onto coverslips at a density of 4 × 10^4^ cells per well. Cells were transiently transfected to coexpress one connexin isoform and one genetically encoded fluorescent sensor:

pDisplay-GRAB_ATP1.0-IRES-mCherry-CAAX was a gift from Yulong Li (Addgene plasmid #167582; http://n2t.net/addgene:167582; RRID: Addgene_167582) (30).

pAEMXT-eLACCO1.1 was a gift from Robert Campbell (Addgene plasmid #167946; http://n2t.net/addgene:167946; RRID: Addgene_167946) (32). To improve expression of eLACCO1.1, this construct was subcloned into the iGluSnFR expression vector backbone. Sequences were verified with Sanger sequencing (GATC).

pCMV(MinDis).iGluSnFR was a gift from Loren Looger (Addgene plasmid #41732; http://n2t.net/addgene:41732; RRID: Addgene_41732) (31).

A mixture of 1 μg of DNA from pCAG-Cx-mCherry construct and 1 μg sensor with 3 μg polyethyleneimine was added to cells for 4 to 8 h. Cells were imaged 48 h after transfection.

### Artificial cerebrospinal fluid

Control (20 mm Hg PCO_2_)—140 mM NaCl, 10 mM NaHCO_3_, 1.25 mM NaH2PO_4_, 3 mM KCl, and 1 mM MgSO4.

Control (35 mm Hg PCO_2_)—124 mM NaCl, 26 mM NaHCO_3_, 1.25 mM NaH_2_PO_4_, 3 mM KCl, and 1 mM MgSO_4_.

Hypercapnic (55 mm Hg PCO_2_)—100 mM NaCl, 50 mM NaHCO_3_, 1.25 mM NaH_2_PO_4_, 3 mM KCl, and 1 mM MgSO_4_.

Hypercapnic (70 mm Hg PCO_2_)—70 mM NaCl, 80 mM NaHCO_3_, 1.25 mM NaH_2_PO_4_, 3 mM KCl, and 1 mM MgSO_4_.

High K^+^ (20 mm Hg)—93 mM NaCl, 10 mM NaHCO_3_, 1.25 mM NaH_2_PO_4_, 50 mM KCl, and 1 mM MgSO_4_. For high K^+^ in 35 mm Hg, the recipe is the same but with 77 mM NaCl.

All solutions had 10 mM d-glucose and 2 mM CaCl_2_ (or MgCl_2_ when [Ca^2+^]_0_ solution was desired) added just before use and saturated with 98% O_2_/2% CO_2_ (20 mm Hg), 95% O_2_/5% CO_2_ (carbogen) (35 mm Hg), or carbogen plus CO_2_ (55 mm Hg and 70 mm Hg). CO_2_ in all solutions was adjusted to give a pH of ∼7.4.

### Live cell fluorescence imaging and analysis

Cells were transiently transfected with one pCAG-Cx-mCherry construct and one genetically encoded fluorescent sensor 48 h prior to imaging. Cells were perfused with control artificial cerebrospinal fluid (aCSF) until a stable baseline was reached before perfusion with either hypercapnic or high K^+^ aCSF. Once a stable baseline was reached after solution change, cells were again perfused with control aCSF, and when a stable baseline was reached, recordings were calibrated by direct application of 3 μM of the corresponding analyte.

All cells were imaged by epifluorescence (Scientifica Slice Scope, Cairn Research OptoLED illumination, 60× water Olympus immersion objective, numerical aperture 1.0, Hamamatsu ImagEM EM-SSC camera; Metafluor software). Circularly permuted GFP (cpGFP) in the sensors was excited by a 470 nm LED, with emission captured between 504 and 543 nm. Connexin constructs have a C-terminal mCherry tag, which is excited by a 535 nm LED, and emission is captured between 570 and 640 nm. Only cells expressing both cpGFP and mCherry were selected for recording, with cpGFP images acquired every 4 s. For each condition, at least three independent transfections were performed with at least two coverslips per transfection.

Analysis of all experiments was carried out in ImageJ (https://github.com/imagej/ImageJ). Images were opened as a stack and stabilized (64). Regions of interest (ROIs) were drawn around cells coexpressing both sensor and connexin. Median pixel intensity was plotted as normalized fluorescence change (ΔF/F_0_) *versus* time to give traces of fluorescence change. The amount of analyte release was quantified as concentration by normalizing to the ΔF/F_0_ caused by application of 3 μM of analyte, which was within the linear portion of the dose–response curve for each sensor. Release from a single cell was considered to be a statistical replicate.

### Confocal imaging of membrane localization

HeLa cells were transfected with mCherry-tagged wildtype and mutant versions of Cx46 and Cx50. After 48 h, the cells were washed three times with PBS, fixed with 4% paraformaldehyde in PBS for 30 min, and then washed three times with PBS. Cells were then incubated with serum-free Dulbecco's modified Eagle's medium containing 2.5 μM DiO (Sigma–Aldrich; catalog no.: D4292) for 15 min. After three washes in PBS, coverslips were mounted inverted on glass microscope slides in a Fluorshield with 4′,6-diamidino-2-phenylindole mounting medium (Sigma–Aldrich; catalog no.: F6057). Slides were sealed and imaged on a Zeiss 880 LSM confocal microscope using the 488 and 561 nm excitation wavelengths for DiO and mCherry, respectively.

Colocalization analysis between the mCherry tag of the connexin variants and the DiO membrane stain was performed with Fiji (https://github.com/fiji/fiji) and the JaCoP plugin (65). ROIs were drawn around mCherry-positive cells, and the image surrounding the ROI was removed. The Manders' coefficient (66) was used as a quantitative assessment of colocalization of mCherry with DiO, and hence, the membrane localization of the connexin constructs. Thresholds were set to a limit that included DiO membrane staining but excluded any diffuse background fluorescence.

### Dye loading

Cells were transiently transfected with pCAG-Cx43-mCherry, pCAG-Cx46^WT^-mCherry, or pCAG-Cx43^QM^-mCherry constructs 48 h prior to experiments. After washing in 20 mm Hg aCSF, cells were perfused with one of the following solutions: 20 mm Hg aCSF, 55 mm Hg aCSF (Cx43 only), Ca^2+^-free aCSF (Cx43 only), or 50 mM KCl aCSF (Cx46^WT^ and Cx46^QM^ only), each containing 50 μM FITC for 10 min. The cells were then washed, first by perfusion with 20 mm Hg in the absence of FITC, before being placed in serial washes of 20 mm Hg aCSF. Cells were fixed in 4% paraformaldehyde for 30 min before being washed three times with PBS. Coverslips were mounted inverted on a microscope slide using Fluorshield with 4′,6-diamidino-2-phenylindole mounting medium (Sigma–Aldrich; catalog no.: F6057). Images were taken using the Zeiss-880 or Zeiss-980 confocal LSM, specifically using the 488 and 561 nm lasers. Subsequent analysis was done using the Fiji software. For Figure 9, figure supplement 1, the median pixel intensity for each transfection was considered to be a statistical replicate.

### Statistical analysis

All quantitative data are presented as box and whisker plots, where the line represents the median, the box is the interquartile range, and the whiskers are the range, with all individual data points included. For multiple comparisons, a Kruskal–Wallis ANOVA was performed, followed by post hoc testing *via* pairwise Mann–Whitney *U* tests (two tailed) with corrections for multiple comparisons using the false discovery method, with a maximum false discovery set to 0.05 (67). In text, all data are presented as median (95% confidence interval, upper and lower limits). All calculations were performed on GraphPad Prism (GraphPad Software, Inc).

## Data availability

All data are available as supporting information within this article.

## Supporting information

This article contains supporting information.

## Conflict of interest

The authors declare that they have no conflicts of interest with the contents of this article.
