# Supplementary material for: Mechanisms of permselectivity of connexin hemichannels to small molecules
Source: J Biol Chem. 2025 Oct 24;301(12):110858. doi: 10.1016/j.jbc.2025.110858 (PMC12666827; doi:10.1016/j.jbc.2025.110858)
Supplement: Supplementary Figures [file mmc1.pdf]

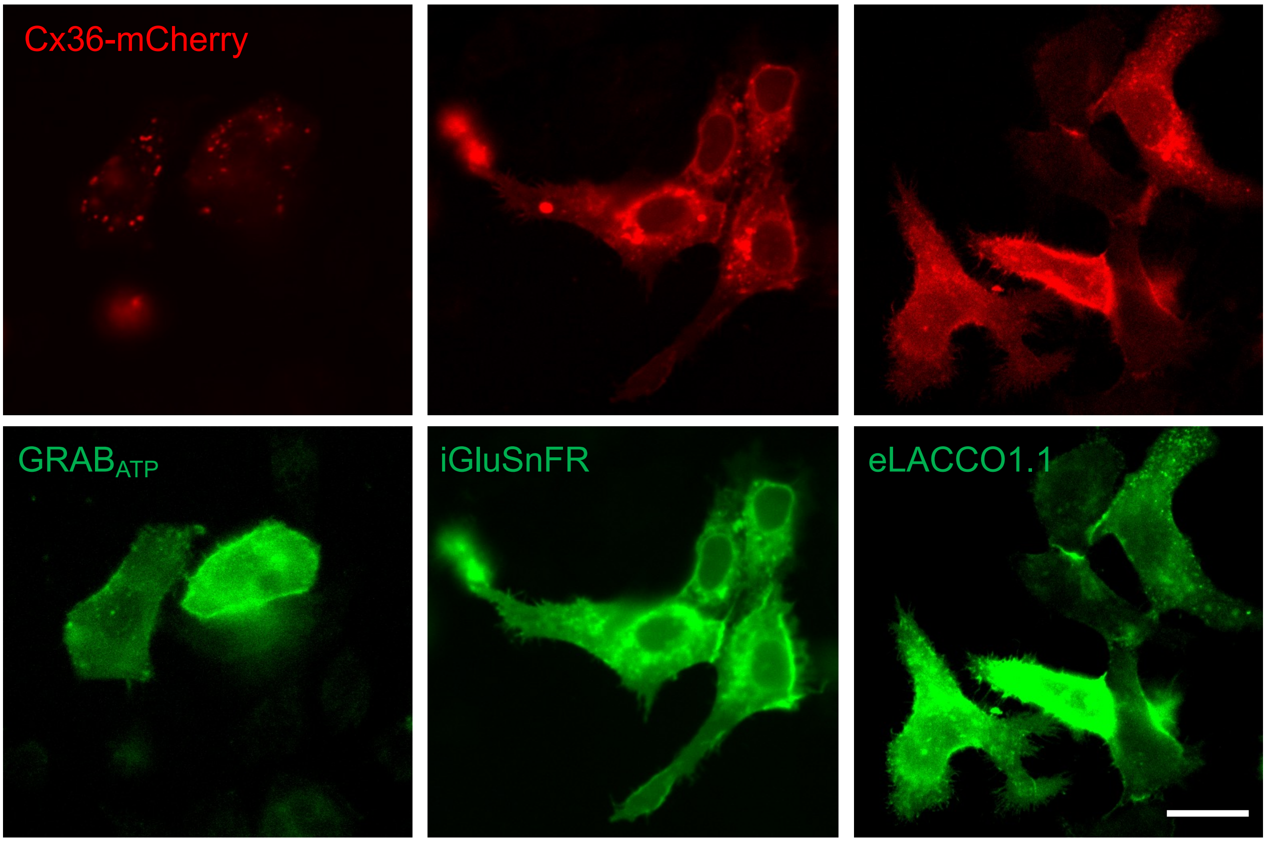

Supplementary Figure 1. Co-expression of Cx-mCherry and sensor (GFP). Using Cx36 as an example, the images demonstrate that cells were selected only if they co-expressed both the connexin construct, which is tagged with mCherry (top row), and the sensor, which is tagged with GFP (bottom row). Scale bar represents 20 μm.

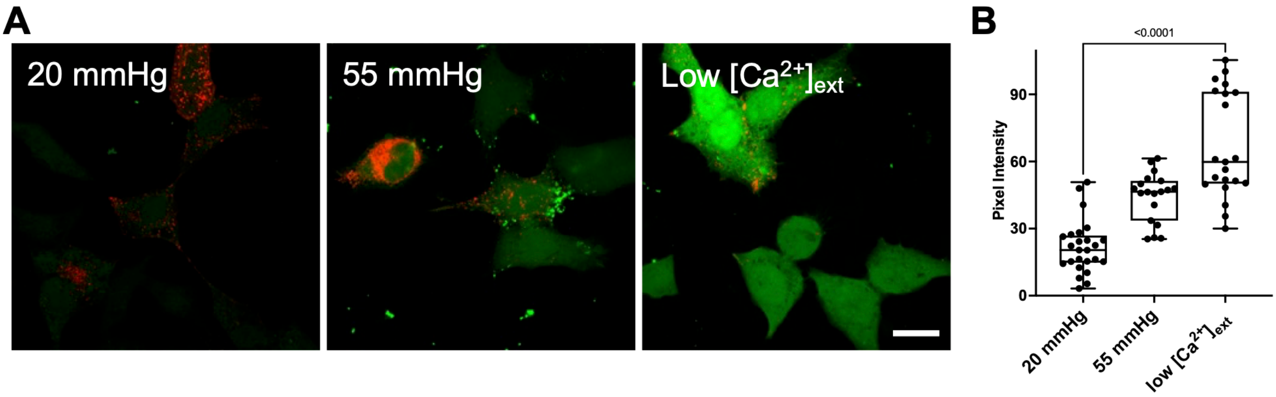

Supplementary Figure 2. Low $[Ca^{2+}]_{ext}$ is sufficient to open Cx43 hemichannels. A. Representative images of FITC loaded into mCherry-tagged Cx43 positive cells. Scale bar 20 μm. **B.** Summary data showing the median pixel intensity under control (20 mmHg) (n = 25), hypercapnia (55 mmHg) (n = 19) and low $[Ca^{2+}]_{ext}$ (n = 21). Mann Whitney *U*-test, $p < 0.0001$ (low $[Ca^{2}]_{ext}$ vs 20 mmHg).

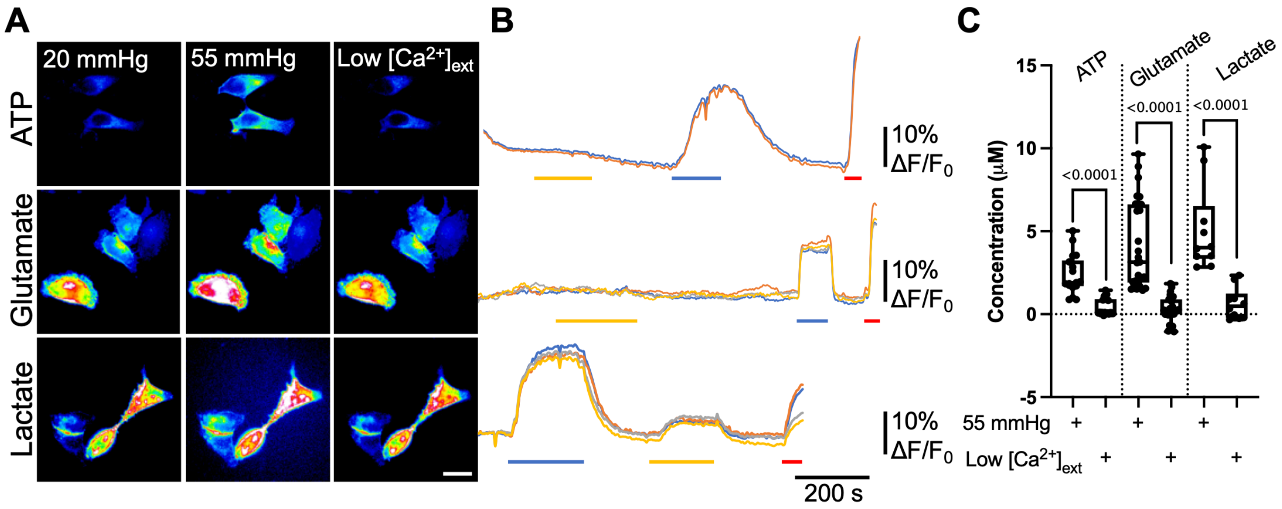

Supplementary Figure 3. Removal of extracellular Ca^{2+} changes the permeability of Cx43 to physiological molecules. A. Representative images showing cells from each sensor under each condition. Scale bar represents 20 μm. **B.** Representative traces of normalised fluorescence changes in response to 55 mmHg pCO_2 (blue bar), low $[Ca^{2+}]_{ext}$ (yellow bar) and a 3 μM calibration of the corresponding analyte (red bar). **C.** Summary data showing the median release of analytes through Cx43 and how it differs depending on the stimulus: ATP (n = 33 for CO_2, n = 17 for high KCl), glutamate (n = 19), lactate (n = 18). Data presented is from at least 3 independent transfections. For 55 mmHg vs low $[Ca^{2+}]_{ext}$ for ATP, glutamate and lactate release, Mann Whitney U-test was performed, $p < 0.0001$.

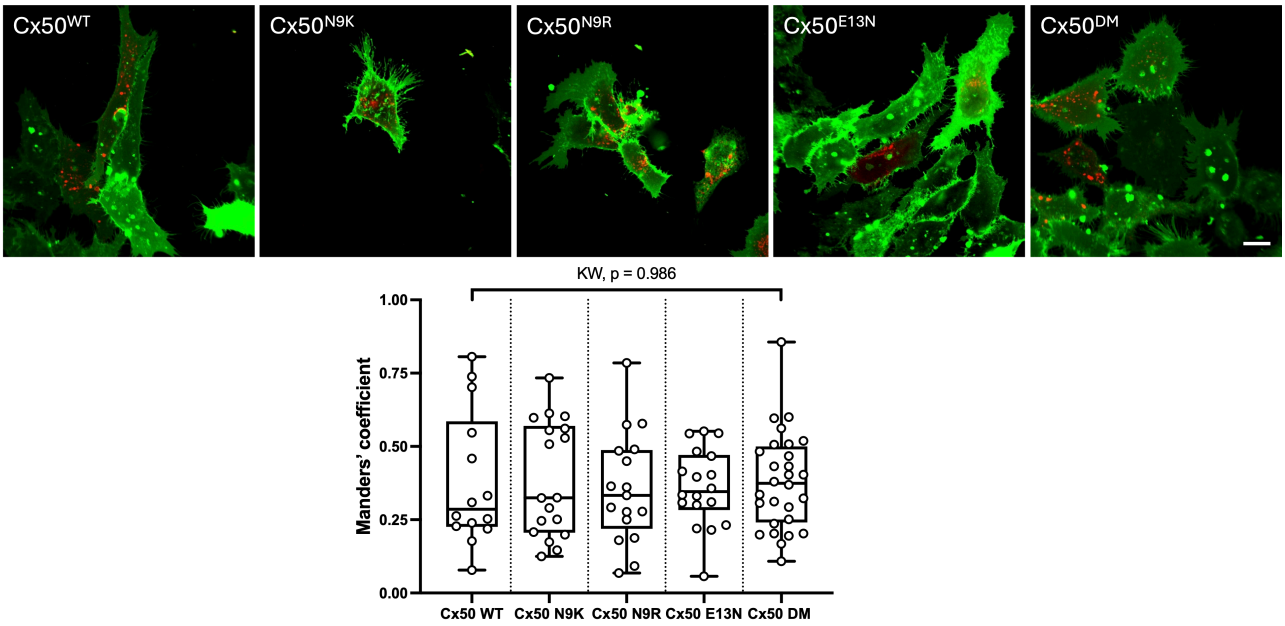

Supplementary Figure 4. The plasma membrane distribution of Cx50 is unaltered by mutations that alter its permeability to ATP. Top, Confocal images of DiO staining (green) and the mCherry-tagged Cx50expression (single optical plane). Scale bar 15 μm. $Cx50^{DM}$ is $Cx50^{N9R\ E13N}$. **Bottom,** Manders' analysis of colocalization shows that the proportion of mCherry colocalised with DiO is the same for $Cx50^{WT}$, and the four mutated mutated variants. Data from 3 independent transfections. Kruskal Wallis Anova, p=0.986
